# Evaluation of Positive and Negative Methods for Isolation of Circulating Tumor Cells by Lateral Magnetophoresis

**DOI:** 10.3390/mi10060386

**Published:** 2019-06-08

**Authors:** Haeli Kang, Jinho Kim, Hyungseok Cho, Ki-Ho Han

**Affiliations:** Department of Nanoscience and Engineering, Center for Nano Manufacturing, Inje University, Gimhae 50834, Korea; rkdgofl9388@naver.com (H.K.); injemems@naver.com (J.K.); elshaddai88@naver.com (H.C.)

**Keywords:** circulating tumor cell, positive isolation, negative isolation, lateral magnetophoresis

## Abstract

We developed an epithelial cell adhesion molecule (EpCAM)-based positive method and CD45/CD66b-based negative method for isolating circulating tumor cells (CTCs) by lateral magnetophoresis. The CTC recovery rate, white blood cell depletion rate, and purity of CTCs isolated using the positive and negative methods were analyzed using blood samples spiked with cancer cells with different expression levels of EpCAM. The aim was to assess the strengths and weaknesses of the positive and negative isolation methods for CTC-based diagnostics, prognostics, and therapeutics for cancer. The EpCAM-based positive method yielded CTCs of high purity, while the CD45/CD66b-based negative method yielded a large number of CTCs. In conclusion, the positive method shows promise for detecting somatic oncogenic mutations and the negative method shows promise for discovery of cellular and transcriptomic biomarkers of cancer.

## 1. Introduction

Circulating tumor cells (CTCs) are an important biomarker for the diagnosis, prognosis, and treatment of cancer [1,2,3]. Despite their clinical importance, there is no standard method for isolating CTCs [4] due to their extreme rarity, typically 1–100 CTCs per 1 mL of peripheral blood [5,6]. Therefore, novel methods for isolating CTCs are required, e.g., the CellSearch system (Menarini Silicon Biosystems), the gold-standard CTC isolation system that was the first to be approved by the United States Food and Drug Administration. Most label-dependent positive isolation methods are dependent on the epithelial cell adhesion molecule (EpCAM; epithelial-specific surface marker) on CTCs [7,8]. Because EpCAM-based positive isolation methods are based on immunoaffinity, they have high selectivity and specificity for isolating CTCs from blood. However, their specificity decreases if CTCs acquire a mesenchymal-like phenotype (including decreased surface expression of EpCAM) following the epithelial–mesenchymal transition and is low for CTCs derived from non-epithelial origin cancers (e.g., melanoma) [9,10,11]. In addition, CTCs expressing mesenchymal markers are related to a poor prognosis [12,13]. Consequently, current EpCAM-based positive isolation methods are inadequate due to the rarity and heterogeneity of CTCs and may miss important subtypes of CTCs [14,15,16].

Negative methods of isolating CTCs from blood involve the removal of white blood cells (WBCs) [17,18,19,20]. Negative methods can be used to harvest all types of CTCs, because they do not depend on the surface-marker profiles or the size of CTCs. In addition, because negative methods do not use antibodies specific to CTCs, they are suitable for downstream analyses, such as genetic assays, CTC culture, and xenografts [21,22]. Several negative methods for isolating CTCs have been developed, but they have lower specificity than positive methods. To date, no study has compared positive and negative microfluidic methods for isolating CTCs under identical conditions.

In this study, we introduce the microfluidic NegCTC-μChip, a negative method of isolating CTCs by lateral magnetophoresis. Anti-CD45/CD66b magnetic nanobeads are used to remove WBCs, which are typically expressed on the membrane of leukocytes and granulocytes, respectively. In addition, we used the previously developed PosCTC-μChip [23] (positive method) to isolate CTCs using anti-EpCAM magnetic nanobeads. We compared the CTC recovery rates, WBC depletion rates, and CTC purity of the PosCTC-μChip and the NegCTC-μChip, using blood samples spiked with cancer cells with different expression levels of EpCAM. Finally, we discussed the strengths and weaknesses of the two methods for CTC-based diagnostics, prognostics, and therapeutics for cancer.

## 2. Materials and Methods

### 2.1. Design and Working Principle

**Figure 1:**
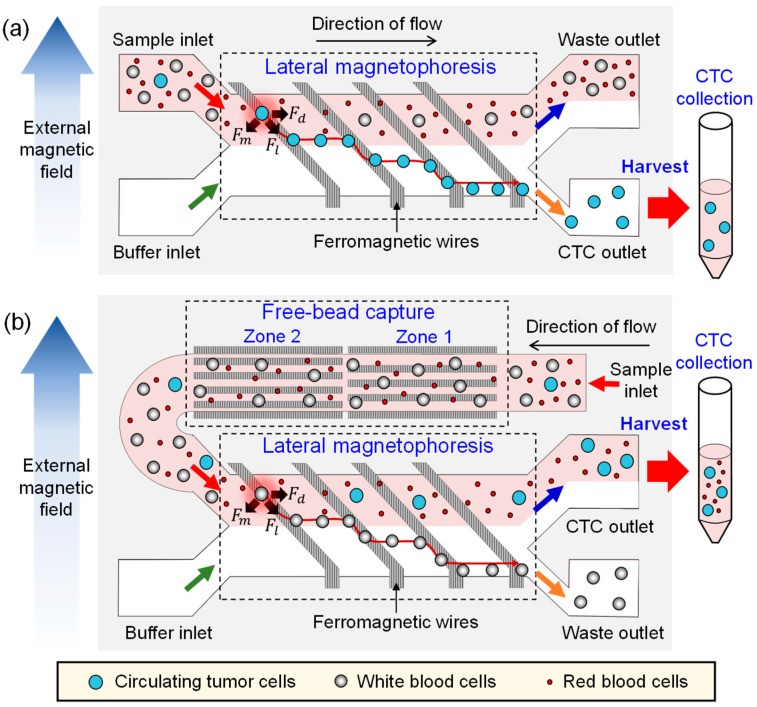
Schematic of (**a**) the PosCTC-μChip, which contains a lateral magnetophoretic microchannel where circulating tumor cells (CTCs) labeled with anti-EpCAM magnetic nanobeads are isolated by lateral magnetophoresis; and (**b**) the NegCTC-μChip, which has a free-bead capture microchannel for removing free magnetic nanobeads and a lateral magnetophoretic microchannel for depleting white blood cells (WBCs) tagged with anti-CD45/CD66b magnetic nanobeads.

The PosCTC-μChip is a microfluidic device for isolating CTCs, based on an immunomagnetic approach that contains a lateral magnetophoretic microchannel where CTCs bound to anti-EpCAM magnetic nanobeads are positively isolated by lateral magnetophoresis (Figure 1a). The NegCTC-μChip consists of a free-bead capture microchannel (for removing free magnetic nanobeads) and a lateral magnetophoretic microchannel (for depleting WBCs bound to anti-CD45/CD66b magnetic nanobeads) (Figure 1b). The lateral magnetophoretic microchannels of both the PosCTC-μChip and the NegCTC-μChip include ferromagnetic wires, which are inlaid into the substrate at 5.7°, relative to the direction of flow. Large numbers of magnetic nanobeads are used in negative isolation methods, because each milliliter of blood contains 5 × 10^6^ WBCs. This results in the presence of unbound residual magnetic nanobeads. As blood flows through the lateral magnetophoretic microchannel, the residual magnetic nanobeads bind to the ferromagnetic wires. Furthermore, DNA strands and serum factors adhere to the stacked nanobeads, and the agglomeration of WBCs and CTCs clogs the microchannel. This phenomenon decreases the recovery rate of CTCs and the depletion of WBCs, which in turn degrades the overall isolation performance. Therefore, residual magnetic nanobeads must be removed prior to isolation of CTCs by negative methods. Because positive methods require fewer magnetic nanobeads, no free-bead capture microchannel is needed. The free-bead capture microchannel is 1 mm in width, 50 μm in height, and 42.5 mm in length to promote capture of residual magnetic nanobeads and to provide stable fluid flow by acting as a fluid impedance, and consists of zones 1 and 2. Because the ferromagnetic wires in the free-bead capture microchannel are parallel to the direction of fluid flow, the nano-sized residual magnetic beads are captured, but micro-sized WBCs are not, thereby preventing microchannel blockage. The ferromagnetic wires in zone 2 are arranged so as to capture any residual magnetic nanobeads not captured in zone 1. The lateral magnetophoretic microchannel of the NegCTC-μChip is 2.8 mm in width and 100 μm in height, which is 5.6-fold larger than the cross-sectional area of the free-bead capture microchannel. Because the buffer solution is injected at a rate equal to that of the blood sample, the mean flow rate is 2.8-fold lower than that of the free-bead capture microchannel. This prolongs WBC exposure to the lateral magnetophoretic force, promoting depletion. To promote isolation of CTCs, the CTC and waste outlets of the NegCTC-μChip are 2.24 and 0.56 mm in width, respectively.

When a uniform external magnetic field is laterally applied to the free-bead-capture and the lateral magnetophoretic microchannels, the external magnetic field near the wires is deformed, generating a high-gradient magnetic field over the microchannels. For the PosCTC-μChip, CTCs are labelled with magnetic nanobeads and behave as paramagnetic particles. When the magnetized CTCs pass over the wire, they simultaneously experience a magnetic force, *F_m_*, and a hydrodynamic drag force, *F_d_*, which generates a lateral magnetic force, *F_l_*, on the CTCs (Figure 1a). Next, the CTCs flow into the CTC outlet and other blood cells flow into the waste outlet. In the case of the NegCTC-μChip, WBCs are tagged with magnetic nanobeads and behave as paramagnetic particles. As a blood sample passes through the free-bead capture microchannel, residual magnetic nanobeads, but not WBCs, are captured on the ferromagnetic wires. Because WBCs are 6−10 µm in diameter, they experience a high drag force and so pass through the free-bead capture microchannel into the lateral magnetophoretic microchannel. As magnetized WBCs pass over the ferromagnetic wires in the lateral magnetophoretic microchannel, they are forced laterally into the waste outlet, while other blood cells (including red blood cells (RBCs) and CTCs) flow into the CTC outlet. Because RBCs lack a nucleus, the sample can be used directly for monitoring CTC populations and for CTC-based genetic analyses.

### 2.2. Fabrication Process

Both the PosCTC-μChip and the NegCTC-μChip were produced by vacuum assembly of a disposable microchannel superstrate and a reusable substrate with inlaid ferromagnetic wires (Figures S1 and S2) [24]. The disposable microchannel superstrate was made by bonding a 12-μm-thick silicone-coated polymer film to a microstructured PDMS replica. An SU-8 mold on a glass master was first fabricated to create a PDMS replica with a microchannel and vacuum trench. An SU-8 3050 photoresist (MicroChem Corp., Westborough, MA, USA) was spun to create the SU-8 mold-pattern for the microchannels on the glass master, and an evaporated 1000 Å Cr layer was applied to promote adhesion between the SU-8 and the glass master. The SU-8 mold pattern for the lateral magnetophoretic microchannel of the PosCTC-μChip was 50 μm in thickness. The free-bead capture and lateral magnetophoretic microchannels of the NegCTC-μChip were 50 and 100 μm in thickness, respectively. Next, an acrylic square bar of 2 × 2 mm^2^ was bonded to the glass master to define the vacuum trench (Figures S1a and S2a). An aluminum mold frame was used to pour the liquid PDMS. The SU-8 mold was completed by assembling the glass master and the aluminum mold frame. Liquid phase PDMS, prepared by mixing resin and curing agent at a 10:1 ratio (Sylgard 184; Dow Corning, Midland, MI, USA), was poured into the SU-8 mold and cured for 1 h at 75 °C in an oven (Figures S1b and S2b). After peeling the PDMS replica off the SU-8 mold, the inlet and outlet reservoirs and the vacuum hole of the PDMS replica were generated using a 1.5-mm-diameter punch. Next, the PDMS replica and the 12-μm-thick silicone-coated polymer film (5 g release force, Shanghai Guangtai Adhesive Products Co., Shanghai, China) were bonded by oxygen plasma treatment for 60 s at 6.8 W radiofrequency power (PDC-32G-2; Harrick Plasma, Ithaca, NY, USA) (Figures S1c and S2c). The reusable substrate was fabricated using a 0.7-mm-thick glass slide (Borofloat33 Pyrex; Schott, New York City, NY, USA). A Ti/Cu/Cr seed layer was first evaporated by electron beam onto the glass slide, and an SU-8 3050 photoresist was spun and patterned to create 40-μm-thick micromolds for ferromagnetic wires (Figures S1d and S2d). Permalloy (Ni_0.8_Fe_0.2_) was electroplated onto the glass slide (Figures S1e and S2e) and 40-μm-thick ferromagnetic wires were formed by chemical and mechanical polishing and inlaid in the reusable substrate (Figures S1f and S2f).

**Figure 2:**
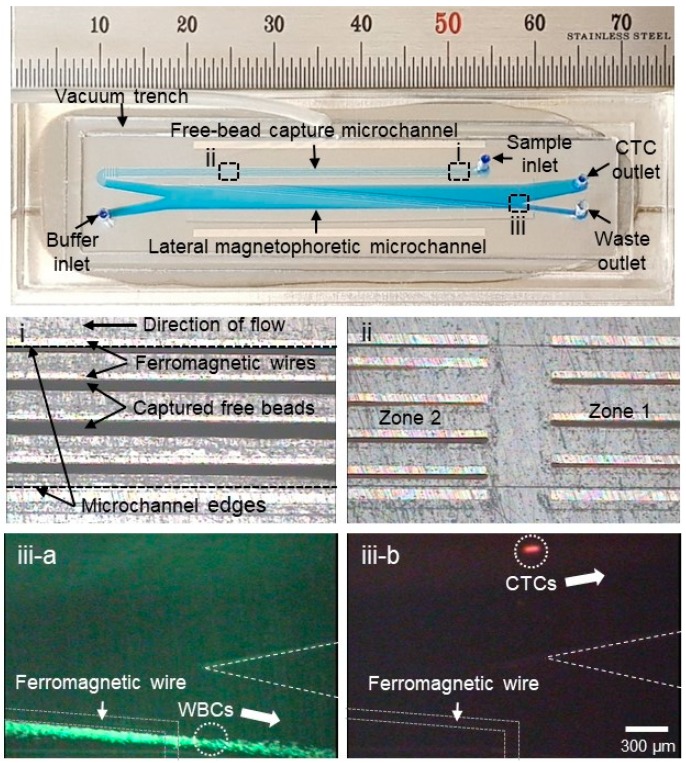
Photographs of the NegCTC-μChip, which consists of free-bead capture and lateral magnetophoretic microchannels. Enlarged views of (**i**) residual magnetic nanobeads captured on the ferromagnetic wires and (**ii**) zones 1 and 2 in the free-bead capture microchannel. In the lateral magnetophoretic microchannel, (**iii**-**a**) WBCs (green) bound to magnetic nanobeads are forced laterally into the waste outlet, and (**iii**-**b**) spiked cancer cells (red) flow into the CTC outlet.

To set up the instrument, the reusable substrate was first placed at the center of two stacked neodymium–iron–boron (Nd–Fe–B) permanent magnets. After alignment of the disposable superstrate to the reusable substrate, an air vacuum pressure of −50 kPa was applied through the vacuum hole to induce bonding of the superstrate to the substrate (Figure 2). The vacuum contact also removed the air gap between the polymer thin film and the ferromagnetic wires, enabling the high-gradient magnetic field generated by the ferromagnetic wires to penetrate the micrometer-thick polymer film into the microchannel and manipulate magnetized CTCs (PosCTC-μChip) or WBCs (the NegCTC-μChip). Two syringe pumps were used to inject the blood sample and the buffer, and another syringe pump was used to suck the solution from the waste outlet to create a stable fluidic flow in the lateral magnetophoretic microchannel. The sample and the buffer flow rates of the PosCTC-μChip were both 2 mL/h and the suction flow rate was 3.2 mL/h, resulting in a flow rate to the CTC outlet of 0.8 mL/h. For the NegCTC-μChip, the sample and the buffer injection flow rates were 2.8 mL/h, to promote the capture of free magnetic nanobeads, but not WBCs, on the ferromagnetic wires and prolong the residence time of WBCs in the lateral magnetophoretic microchannel, promoting their depletion. A high flow rate hampers depletion of WBCs, due to the low capture efficiency of residual magnetic nanobeads in the free-bead capture microchannel and the reduced residence time of WBCs in the lateral magnetophoretic microchannel. A low flow rate results in capture of WBCs in both the free-bead capture and the lateral magnetophoretic microchannels, decreasing the CTC yield. CTCs and WBCs in the CTC outlet were enumerated under a fluorescence microscope and the CTC recovery rate, WBC depletion rate, and CTC purity were calculated. 

### 2.3. Sample Preparation

Human peripheral blood was obtained from healthy donors, using a protocol approved by the institutional review board of Inje University (Inje 2017-05-013-003), collected in Vacutainer tubes (G-Tube™, Green Cross, Yongin, Korea) containing anticoagulant ethylenediaminetetraacetic acid, and processed within 6 h. MDA-MB-231 and MCF-7 cells were cultured in Dulbecco’s Modified Eagle’s Medium (DMEM, Invitrogen, Waltham, MA, USA), whereas PC-3 and SKBR-3 cells were grown in RPMI-1640 culture medium (Invitrogen) in a 95% humidified atmosphere and 5% CO_2_ at 37 °C. Both media contained 10% (v/v) fetal bovine serum, 100 units/mL penicillin, and 1 mg/mL insulin. Prior to use in experiments, they were harvested from culture plates, using 0.25% trypsin at 37 °C for 3 min and transferred to 15 mL conical tubes. The harvested cancer cells were stained with a membrane-permeable red fluorescent nucleic acid dye (SYTO 64, Invitrogen) for 10 min at room temperature. The fluorescently stained cancer cells were washed in phosphate-buffered saline (PBS) with 0.2% bovine serum albumin (BSA) and suspended to a density of approximately 10^4^ per mL. Non-tumor cells in 500 μL of healthy whole blood were stained with a membrane-permeable green fluorescent nucleic acid dye (SYTO 13, Invitrogen). Next, 100 fluorescent-stained CTCs were spiked into the 500-μL blood sample in a 1.5-mL microcentrifuge tube. The spiked blood sample was added to antibodies (anti-EpCAM antibody (Human EpCAM Positive Selection Kit, STEMCELL Technologies, Vancouver, BC, Canada) for the PosCTC-μChip and anti-CD45 and -CD66b antibodies (EasySep Human CD45 Depletion Kit and EasySep Human Whole Blood CD66b Positive Selection Kit, STEMCELL Technologies) for the NegCTC-μChip) and magnetic nanobeads (about 50 nm in diameter) in sequence and incubated on ice for 60 and 90 min, respectively, according to the manufacturer’s instructions (STEMCELL Technologies). During incubation, the antibodies bound to target cells and magnetic nanobeads, because they were bispecific to combine surface antigens expressed on target cells and dextran coated on magnetic nanobeads [25]. Finally, the sample was diluted with a fourfold volume (2 mL) of ice-cold PBS containing 0.2% BSA.

## 3. Results and Discussion

To evaluate the performance of the PosCTC-μChip and the NegCTC-μChip, MDA-MB-231, PC-3, SKBR-3, and MCF-7 cancer cells were used. The EpCAM expression levels of the cancer cell lines were quantified by flow cytometry (FACSCalibur, BD Biosciences, Franklin Lakes, NJ, USA), using a fluorescein isothiocyanate-labeled anti-EpCAM antibody (anti-human CD326, BioLegend) (Figures S3 and S4). The CTC recovery rate, WBC depletion rate, and CTC purity were calculated using the following equations:

(1) $$\mathrm{CTC}\;\mathrm{recovery}\;\mathrm{rate}\;\left(\%\right)=\frac{\mathrm{The}\;\mathrm{number}\;\mathrm{of}\;\mathrm{isolated}\;\mathrm{cancer}\;\mathrm{cell}\;\mathrm{lines}}{\mathrm{The}\;\mathrm{number}\;\mathrm{of}\;\mathrm{spiked}\;\mathrm{cancer}\;\mathrm{cell}\;\mathrm{lines}}\times 100$$

(2) $$\mathrm{WBC}\;\mathrm{depletion}\;\mathrm{rate}\;(\mathrm{Log})\;=\;\log\left(\frac{\mathrm{The}\;\mathrm{initial}\;\mathrm{number}\;\mathrm{of}\;\mathrm{WBCs}}{\mathrm{The}\;\mathrm{number}\;\mathrm{of}\;\mathrm{contaminated}\;\mathrm{WBCs}}\right)$$

(3) $$\mathrm{CTC}\;\mathrm{purity}\;\left(\%\right)\;=\frac{\mathrm{The}\;\mathrm{number}\;\mathrm{of}\;\mathrm{isolated}\;\mathrm{cancer}\;\mathrm{cell}\;\mathrm{lines}}{\mathrm{The}\;\mathrm{number}\;\mathrm{of}\;\mathrm{isolated}\;\mathrm{total}\;\mathrm{nucleated}\;\mathrm{cells}\;(\mathrm{cancer}\;\mathrm{cell}\;\mathrm{lines}\;\mathrm{and}\;\mathrm{WBCs})}\times 100$$

CTCs and WBCs were enumerated by counting red-fluorescent and green-fluorescent cells, respectively. The number of isolated total nucleated cells is the sum of the number of isolated CTCs and WBCs. We confirmed the number of spiked CTCs by counting those in the CTC outlet and in the sample syringe, microfluidic device, and waste outlet.

**Figure 3:**
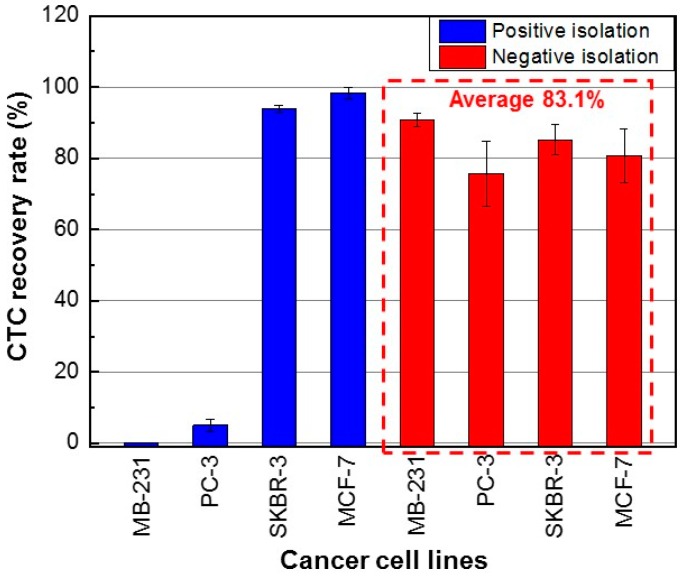
CTC recovery rates using the PosCTC-μChip and NegCTC-μChip from 500-μL blood samples spiked with CTCs with different expression levels of EpCAM. The data are means of triplicate determinations.

The CTC recovery rate, WBC depletion rate, and CTC purity were evaluated using three measurement datasets. Using the PosCTC-μChip, the recovery rates of SKBR-3 and MCF-7 cells (high EpCAM expression) were 93.9 ± 1.0% (mean ± SD) and 98.4 ± 1.5%, respectively (Figure 3). In contrast, the recovery rates of MDA-MB-231 and PC-3 cells (low EpCAM expression) were 0% and 5.1 ± 1.7%, respectively. Thus, the EpCAM-based positive method had limited ability to isolate CTCs with low EpCAM expression. Using the NegCTC-μChip, the recovery rates of SKBR-3 and MCF-7 cells were 85.2 ± 4.2% and 80.7 ± 7.6%, respectively, and those of MDA-MB-231 and PC-3 cells were 91.0 ± 2.0% and 75.7 ± 9.3%, respectively. Therefore, the NegCTC-μChip enabled isolation of CTCs with a mean recovery rate of 83.1%, irrespective of their EpCAM expression level (Figure 3). CTC recovery rates of the negative isolation method showed greater variations than those of the positive isolation method, because spiked cancer cells could easily be damaged during the sample preparation procedure, causing non-specific binding with magnetic nanobeads.

**Figure 4:**
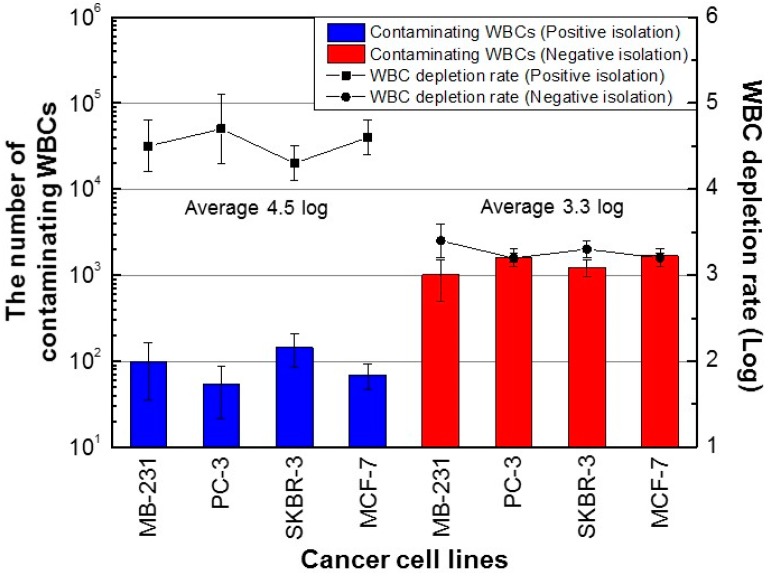
Number of contaminating WBCs using the PosCTC-μChip and the NegCTC-μChip from 500-μL blood samples spiked with CTCs. Data are means of triplicate determinations.

For the positive isolation method, the number of contaminating WBCs was 22‒213 per 500 μL of blood (Figure 4). The mean number of contaminating WBCs was 92; thus, based on a value of 2.5 × 10^6^ WBCs per 500 μL of blood, the average WBC depletion rate was 28,261-fold (4.5 log). For the negative isolation method, the number of contaminated WBCs was 605‒1830 per 500 μL of blood (Figure 4). The mean number of contaminating WBCs was 1379 cells, for a mean WBC depletion rate of 1813-fold (3.3 log). Therefore, some WBCs were not labeled with anti-CD45/CD66b magnetic nanobeads, suggesting the presence of WBC subtypes with low CD45 and CD66b expression.

**Figure 5:**
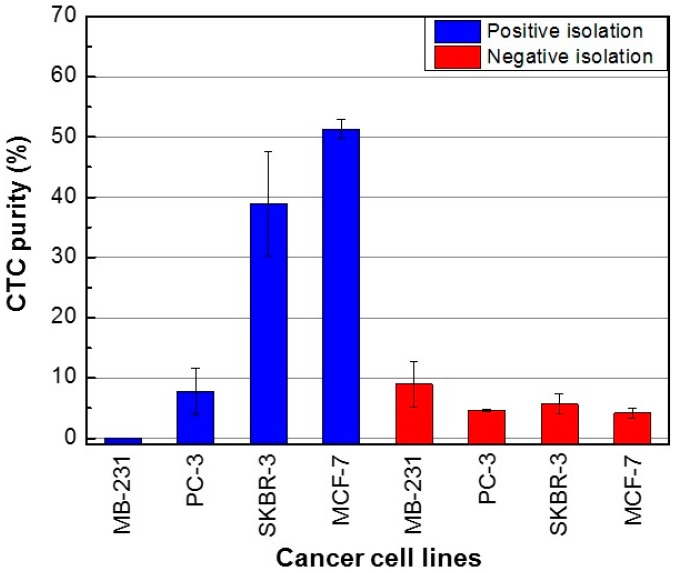
Purity of cancer cells isolated using the PosCTC-μChip and the NegCTC-μChip from 500-μL blood samples spiked with the CTCs. Data are means of triplicate determinations.

We calculated the purity of the CTCs isolated using the PosCTC-μChip and the NegCTC-μChip as the ratio of the number of CTCs to the sum of the number of CTCs and WBCs (Equation (3)). Using the positive method, the purities of SKBR-3 and MCF-7 cells (high EpCAM expression) were 38.9 ± 8.7% and 51.3 ± 1.6%, respectively (Figure 5). In contrast, the purities of MDA-MB-231 and PC-3 cells were 0% and 7.7 ± 3.9%, likely due to their low expression level of EpCAM. CTC purities of the positive isolation method showed large variations, because the number of contaminating WBCs showed a significant variation, despite being 10 times lower than that of the negative isolation method. Using the negative method, the purities of MDA-MB-231, PC-3, SKBR-3, and MCF-7 cells were 9.0 ± 3.8%, 4.6 ± 0.2%, 5.7 ± 1.7%, and 4.2 ± 0.4%, respectively. Despite the high recovery rate, the purity of CTCs isolated using the NegCTC-μChip was insufficient, due to the number of contaminating WBCs.

## 4. Conclusions

We compared an EpCAM-based positive isolation method and a CD45/CD66b-based negative isolation method, using MDA-MB-231, PC-3, SKBR-3, and MCF-7 cancer cells, which have different surface expression levels of EpCAM. The recovery rates of the positive isolation method increased with increasing EpCAM expression level. In contrast, the negative method enabled isolation of cancer cells from blood samples with an average recovery rate of 83.1%, irrespective of the expression level of EpCAM. Therefore, the negative isolation method yielded a larger quantity of CTCs from blood samples than the positive method, irrespective of their EpCAM expression level. WBCs were also extracted from the blood samples using the positive and negative isolation methods. In the case of the positive isolation method (mean, 92 WBCs per 500 µL of blood), this may have been due to non-specific binding of WBCs to anti-EpCAM magnetic nanobeads. In the case of the negative isolation method, the contaminating WBCs (mean 1379; 10-fold increase compared to the positive isolation method) were those not bound to anti-CD45/CD66b magnetic nanobeads, due to their abnormally low expression of CD45 and CD66b. Of note, the number of contaminating WBCs was similar in both the positive and negative isolation methods (Figure 4). The results of CTC recovery rate and WBC depletion rate are in line with studies reported by the Toner group [17], although they did not use the same cancer cells for positive and negative isolation. 

Regarding the purity of isolated CTCs, because the positive method showed a relatively constant number of contaminating WBCs, CTC purity increased with increasing EpCAM expression level. In the case of the negative method, because the numbers of isolated CTCs and WBCs were similar, irrespective of the EpCAM expression level of the former, CTC purity was 4.2‒9.0%. The purity of isolated CTCs was higher with the positive method than with the negative isolation method, with the exception of MDA-MB-231 (low EpCAM expression), due to the larger number of contaminating WBCs using the latter method. Our results indicate that the positive isolation method can be used to obtain highly pure CTCs, enabling detection of somatic oncogenic mutations using advanced genetic analysis techniques (such as next-generation sequencing) that require genomic DNA samples of >5% purity [26,27]. The negative isolation method has a higher yield than the positive isolation method, making it suitable for discovery of cellular and transcriptomic biomarkers of cancer. Therefore, the isolation method suitable for the application in question should be selected.

## Supplementary Materials

The following are available online at https://www.mdpi.com/2072-666X/10/6/386/s1, Figure S1: Fabrication process for the PosCTC-μChip, Figure S2: Fabrication process for the NegCTC-μChip, Figure S3: EpCAM expression level of cancer cell lines used.
