# Supplementary material for: Evaluation of Positive and Negative Methods for Isolation of Circulating Tumor Cells by Lateral Magnetophoresis
Source: Micromachines (Basel). 2019 Jun 8;10(6):386. doi: 10.3390/mi10060386 (PMC6631028; doi:10.3390/mi10060386)
Supplement: Supplementary file 1 [file micromachines-10-00386-s001.pdf]

Supplementary Materials: Evaluation of Positive and Negative Methods for Isolation of Circulating Tumor Cells by Lateral Magnetophoresis

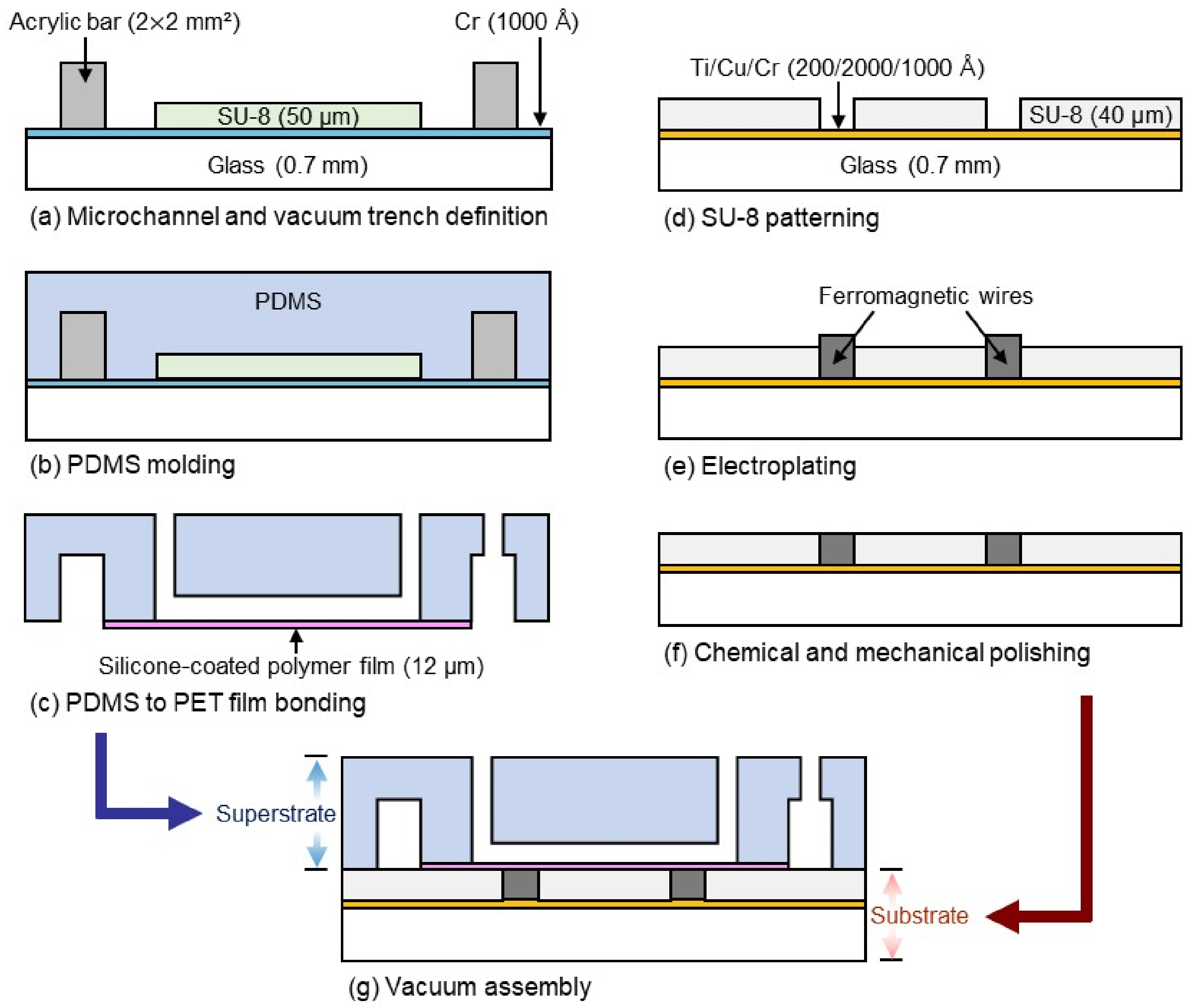

Figure S1. Fabrication of the PosCTC-μChip. (a) Cr (1000 Å) deposition on a glass slide, SU-8 patterning to define the microchannel, and the adhesive-bonded acrylic square bar (2 × 2 mm²) that defines the vacuum trench. (b) PDMS molding to create a microstructured PDMS replica. (c) Inlets, outlets, and a vacuum hole were created using a 1.5-mm-diameter punch and oxygen plasma bonding of the PDMS replica and a silicone-coated 12-μm-thick polymer film was performed to produce the disposable microchannel superstrate. (d) Deposition of Ti/Cu/Cr (200/2000/1000 Å) on a glass substrate and SU-8 patterning to create a 40-μm-thick micromold. (e) Permalloy ($Ni_{0.8}Fe_{0.2}$) electroplating of ferromagnetic wires. (f) Chemical and mechanical polishing of the permalloy to create inlaid ferromagnetic wires, completing fabrication of the reusable substrate. (g) Vacuum assembly of the disposable microchannel superstrate and the reusable substrate to create the PosCTC-μChip.

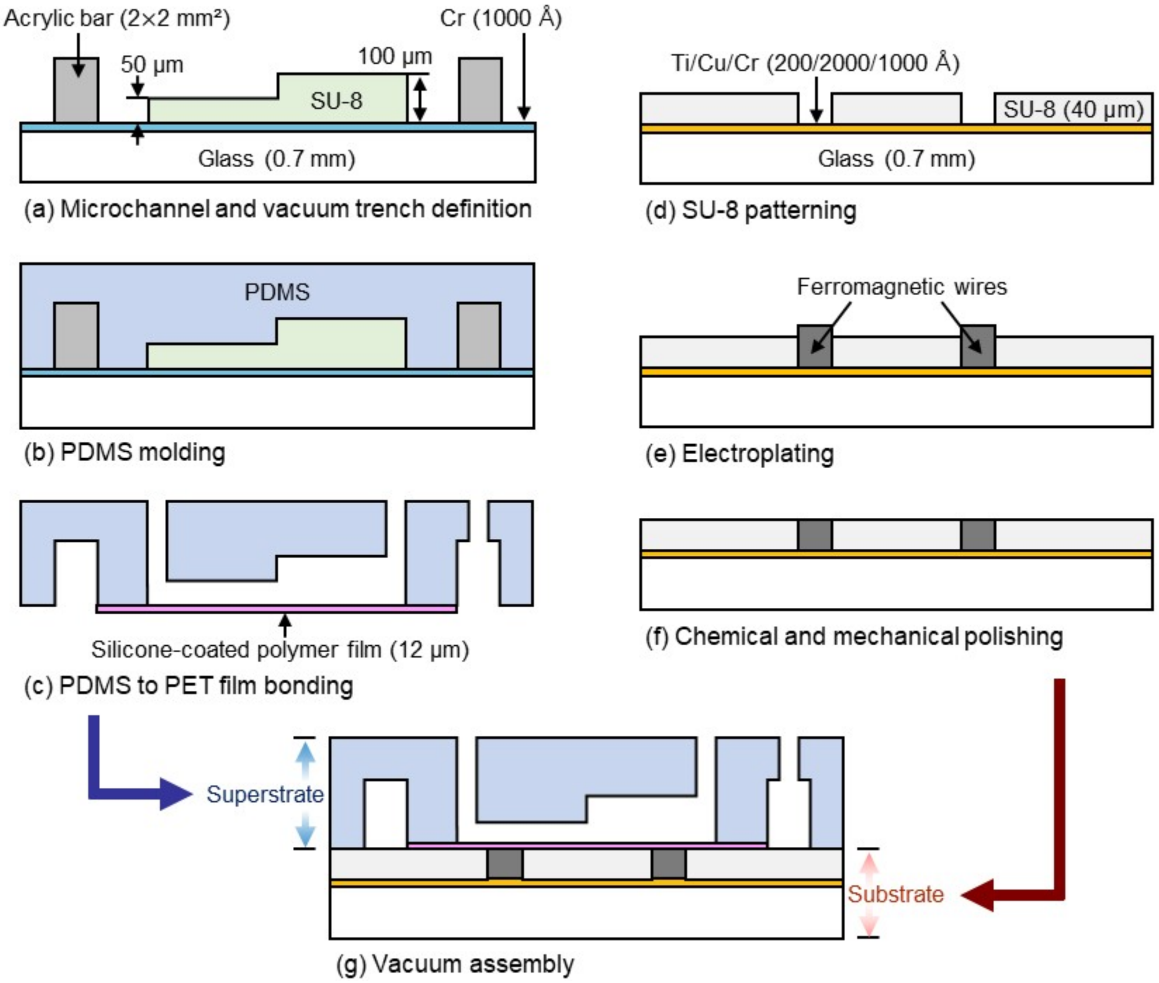

Figure S2. Fabrication of the NegCTC-μChip was identical to that of the PosCTC-μChip, except that the SU-8 patterns that enable free-bead capture and the lateral magnetophoretic microchannels were 50 and 100 μm in height, respectively.

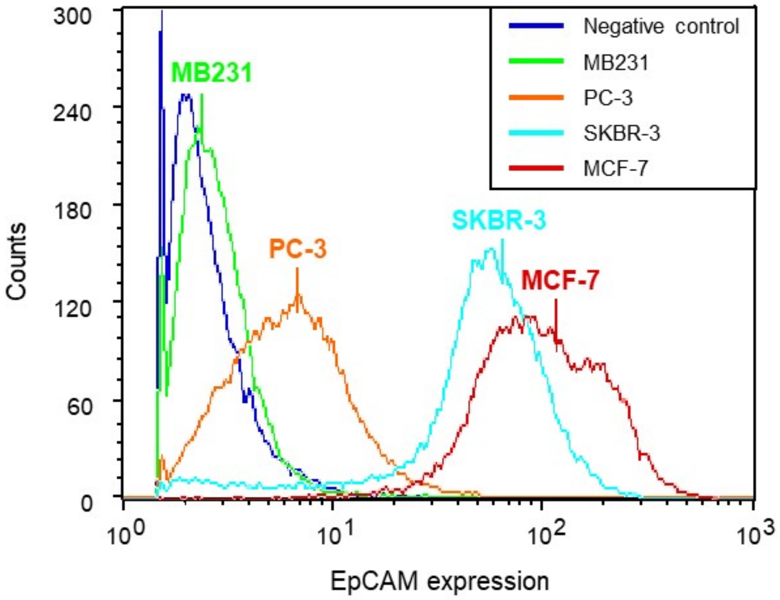

Figure S3. EpCAM expression levels of MDA-MB-231, PC-3, SKBR-3, and MCF-7 cells determined by flow cytometry (FACSCalibur, BD Biosciences).

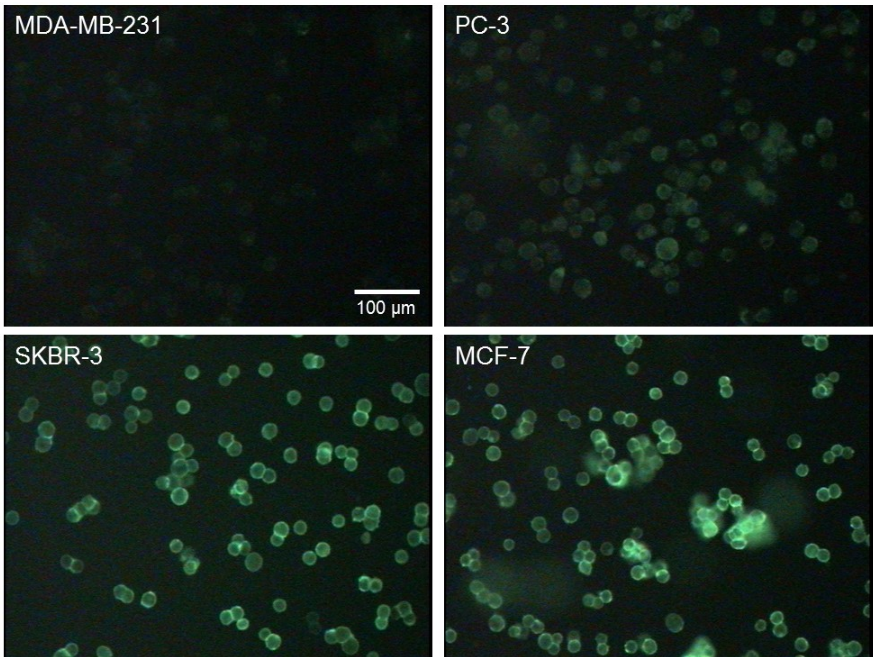

Figure S4. Fluorescence images of MDA-MB-231, PC-3, SKBR-3, and MCF-7 cells stained by a fluorescein isothiocyanate-labeled anti-EpCAM antibody.
